# High Biological Value Compounds Extraction from Citrus Waste with Non-Conventional Methods

**DOI:** 10.3390/foods9060811

**Published:** 2020-06-20

**Authors:** Mayra Anticona, Jesus Blesa, Ana Frigola, Maria Jose Esteve

**Affiliations:** Nutrition and Food Chemistry, University of Valencia, Avda., Vicent Andrés Estellés, s/n., 46100 Burjassot, Spain; Mayra.Anticona@ext.uv.es (M.A.); Jesus.blesa@uv.es (J.B.); ana.frigola@uv.es (A.F.)

**Keywords:** citrus waste, high-biological-value compounds, extraction, non-conventional methods

## Abstract

Citrus fruits are extensively grown and much consumed around the world. Eighteen percent of total citrus cultivars are destined for industrial processes, and as a consequence, large amounts of waste are generated. Citrus waste is a potential source of high biological value compounds, which can be used in the food, pharmaceutical, and cosmetic industries but whose final disposal may pose a problem due to economic and environmental factors. At the same time, the emerging need to reduce the environmental impact of citrus waste and its responsible management has increased. For these reasons, the study of the use of non-conventional methods to extract high biological value compounds such as carotenoids, polyphenols, essential oils, and pectins from this type of waste has become more urgent in recent years. In this review, the effectiveness of technologies such as ultrasound assisted extraction, microwave assisted extraction, supercritical fluid extraction, pressurized water extraction, pulsed electric field, high-voltage electric discharges, and high hydrostatic pressures is described and assessed. A wide range of information concerning the principal non-conventional methods employed to obtain high-biological-value compounds from citrus waste as well as the most influencing factors about each technology are considered.

## 1. Introduction

Citrus fruits, which belong to the Rutaceas family, are among the most commonly grown and consumed fruits around the world [1]; the production of citrus fruits in 2015 was more than 130 million tons [2]. Among these are mainly oranges, mandarins, limes, lemons, and grapefruits [3], and citron and bergamot are also grown [4]. Of the total citrus fruits grown worldwide, 18% are destined for industrial processes [2], mainly for the production of juice [5]. However, the creation of industrialized citrus products generates large amounts of waste (peel, pulp, and seed residues), between 50% and 70% of the total citrus fruit is destined for this activity [5,6,7]. Approximately 15–25 thousand tons of citrus waste is produced annually, which is a potential source for various beneficial uses [8]. On the other hand, the final disposal of citrus waste generates pollution problems [5], especially in developing countries, and its management has a high cost in economic and environmental terms [5,9,10]. The high level of pollution of citrus waste is due to its easy fermentability [11], as citrus waste is bulky, heterogeneous, chemically complex, and highly biodegradable, with a high chemical oxygen demand (for example, 1085 mg of O2/g in the case of orange peel) [12]. Among other characteristics, these wastes have a low pH (3–4), and a high content of water (80–90%) and organic matter (95% of total solids) [13]. Directive (EU) 2018/851 [14] of the European Parliament and of the Council states that for citrus residues, like any other type of food waste, recovery and adequate management and/or safe disposal must be guaranteed due to their biodegradable characteristics. These regulations were established in order to protect human health and reduce environmental and economic concerns about the high cost of disposal [15]. Losses and disposal of this type of waste also indirectly include the waste of critical resources such as land, water, fertilizers, chemicals, energy, and labor [16].

For years, the main use of citrus residues has been pectin extraction or animal feed [17], compost production [18], as well as fuel production [19]; however, in some cases this can be rather costly [12]. Therefore, alternatives for the most adequate and eco-sustainable management of this type of waste have been proposed, such as its use for the production of nanocellulose [20], pectinase [21,22], larvicides [23], and bioethanol [24], biofuels [8,25,26,27,28], including nanoparticles of iron [29] and silver, with cytotoxic activity [30].

Citrus waste has a high biological value with potential health benefits [31] due to its high-quality fiber, pectin content, and the presence of bioactive compounds such as polyphenols (flavonoids and phenolic acids), carotenoids, and essential oils (EOs) [3,32,33,34,35], which can be used in the food, pharmaceutical, and cosmetics industries [9,36]. Recently some “not peer reviewed” articles showed the relationship of citrus hesperidin and COVID-19 disease. This is based on the performs of hesperidin and its interaction to receptors SARS-CoV-2 main protease (PDB:6Y84) [37] and crystal structure main protease (PDB:6LU7), Spike glycoprotein-RBD (PDB:6LXT), and PD-ACE2 (PDB:6VW1) [38], which could have an inhibitory effect against virus infection and replication [37,38]. Since hesperidin is abundant in citrus peel, several studies presented citrus waste as a good option to obtain this compound from a natural source. Based on the interactions with receptors of SARS-CoV-2, clinical trials should be carried out with this product to establish its prophylactic or therapeutic activity against COVID-19 [39,40].

Conventional methods, employing organic solvents, require long extraction times, large amounts of energy and other resources, and therefore pose as great an environmental risk [41] as the biodegradable characteristics of citrus waste already do [12]. The interest in the responsible management of this type of waste has increased because of the drawbacks of conventional extraction methods, the wide availability of citrus waste, and the emerging need to reduce the environmental impact [10]. Thus, the study of non-conventional technologies known as “green extraction methods” has gained relevance [7]. These are the best sustainable options for obtaining bioactive compounds from citrus residues [34,42], providing a better use of these resources. An improved performance; lower expenditure of energy, time, and other resources; and the minimum or zero use of organic solvents are their main advantages [41,43]. In this context the EU proclaimed, “to contribute to the achievement of the United Nations Sustainable Development Goals and ensure that the appropriate path of waste reduction is followed at Union level, of 30% by 2025 and 50% by 2030” [14]. These novel methods include ultrasonic extraction, microwave-assisted extraction, supercritical fluid extraction, pressurized water extraction, electrical pulses, electrical discharges, high hydrostatic pressure, etc. In this line, recent advances in the use of natural solvents to extract bioactive compound have been published. Deep eutectic solvents (DESs) are the most sustainable option to extract natural components from food byproduct sources [44,45]. However, the study of its use in citrus waste has to be explored in depth. Also, the application of non-conventional methods employing DESs is a promising research line.

**Figure 1:**
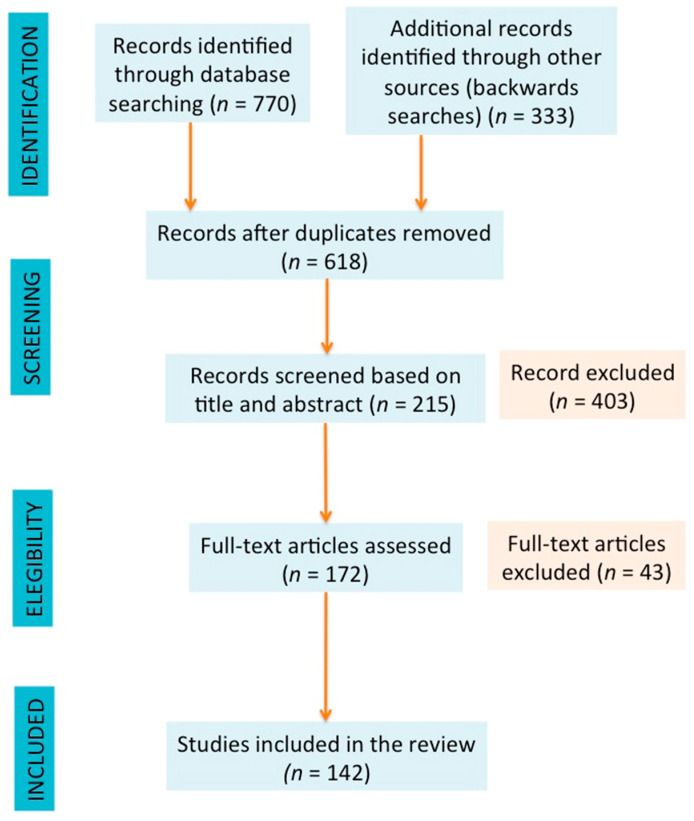
Bibliographic flow chart (adapted from Moher et al. [46]).

This review includes the main findings regarding the extraction of high biological value compounds (carotenoids, polyphenols, essential oils, and pectins) from citrus waste extracted by non-conventional treatments based on systematically searching the scientific literature using databases such as SCOPUS, Web of Science, and Google Scholar using the keywords “citrus” AND “waste” AND “high biological value compounds” AND “extraction” AND “non-conventional.” The literature was classified according to the non-conventional methods used and the bioactive compounds extracted, prioritizing the articles published from 2010 to 2020; however, some articles published in previous years (one published in 1992, four published in 2006, one published in 2007, and three published in 2009) were included in the review approach. Books and reviews were included in the selected literature. Only full-text English articles were considered. Figure 1 shows a flow chart summarizing the selection of articles included in the review.

The aim of this review is to summarize, discuss, and provide information on the non-conventional technologies and methods that have been developed to improve the sustainable derivation of high-value biological compounds such as carotenoids, flavonoids, phenolic acids, essential oils, and pectin from citrus waste.

## 2. Principal Non-Conventional Technologies to Extract High Biological Value Compounds from Citrus Waste

### 2.1. Ultrasound Assisted Extraction

Ultrasonic application, commonly known as ultrasound assisted extraction (UAE), is a non-conventional technology that uses waves with a frequency above 10 MHz [47]. At first, its application was focused on food preservation, but in the last decade, it has also been used for the extraction of bioactive compounds (mainly polyphenols). Due to the ease of the process, advantages such as reduction of extraction time, increase in extract yield, use of water as a solvent reducing the use of organic solvents have been described, which reduce environmental risks. For these reasons, UAE is considered a “green extraction method” [48].

Ultrasonic waves can alter cell walls, since some are strong enough to form bubbles in the liquid, which collapse and increase the interaction between the solvent and the compounds present in the samples; this phenomenon is known as cavitation [49,50]. The collapse caused produces enough energy for chemical reactions, so extractions made by ultrasounds (USN) are of higher performance and quality than those obtained by conventional methods [51].

The cavitation and disruptive properties of USN have been used for the extraction of bioactive compounds and pectins from fruit residues such as citrus fruits [52]. Several studies detail the extraction of carotenoids, phenolic compounds, and pectins from citrus waste by USN, as well as the influence of the used parameters (Table 1). In most USN studies, the optimization of the method and/or a study design is included. In this line, the response surface methodology (RSM) is very useful.

In the extraction of carotenoids from citrus waste, the matter used for extraction is mainly the peel, since carotenoids, being natural fat-soluble pigments, are responsible for the yellow, orange, and red colors in citrus fruits, in addition to participating in the processes of photosynthesis and photoprotection. Its structure is composed of bonds (double and single) of 40 terpenoid carbons constructed from isoprenoids linked by the center of the molecule [79,80]. Due to this structure, some kinds of carotenoids are found more related to polar solvents such as acetone, or to a-polar solvents such as hexane. These types of solvents are highly toxic and difficult to eliminate. In contrast, “green” alternatives such as the UAE treatment are proposed for the extraction of carotenoids using environmentally friendly solvents. For instance, Sun et al. [53] found that the use of ethanol as a solvent favored greater extraction with UAE compared to conventional extraction (CE). Thus, ethanol is an efficient alternative to a-polar solvents for the extraction of carotenoids with UAE. Another alternative was reported by Boukroufa et al. [55], who achieved a high yield of carotenoids in orange peel extract using UAE and limonene (previously extracted) as the solvent in a short extraction time (5 min). The carotenoid concentration was 40% higher with UAE than CE with hexane as the solvent.

A new alternative to the use of traditional solvents was proposed by Murador et al. [57]. They published a method to extract carotenoids from orange peel by UAE using ionic liquids (ILs) as solvents. They observed that the use of ILs favored the yield of the extract with ultrasound for 5 min compared to acetone and conventional methods. In their study, 3.21 mg β-carotene/100 g of total carotenoids was recovered with chlorinated 1-Butyl-3-methylimidazolium (BMIM-Cl), in contrast to the 0.78 mg/100 g of total carotenoids recovered with acetone.

Recently, Saini, Panesar, and Bera [54] carried out a study to optimize the parameters in the treatment with UAE for the extraction of lutein from kinnow mandarin (*Citrus reticulata*) peel. The authors determined that temperature, time, and amplitude significantly affected (*p* < 0.05) the lutein yield. With optimal parameters, a maximum lutein yield (2.97 mg/100 g) similar to the predicted design yield (2.85 mg/100 g) was obtained. A similar study was carried out by Montero-Calderón et al. [56], who applied ultrasound treatment for the extraction of total carotenoids (among other bioactive compounds) from orange peel (*Citrus sinensis*) grown in Valencia (Spain). They recovered 0.63 mg of β-carotene/100 g of peel using ethanol 50% during 30 min, similar to the predetermined value (0.52 mg β-carotene/100 g of peel) using that design. In both cases, RSM was used for parameter optimization.

In relation to phenolic compounds, some studies detail the effectiveness of the extraction of these compounds from citrus residues using UAE. Phenolic compounds are the major secondary metabolites in plants, and there are more than 9000 recognized chemical structures [81,82,83,84]. Flavonoids and phenolic acids are the main polyphenols in citrus species [85]. However, flavonoids are the major phenolic compounds presents in citrus peel [34]. The effectiveness of UAE against CE and the parameters analyzed to recover phenolic compounds from citrus waste are described in several studies. For instance, Ma et al. [63] considered in their study that time, temperature, and the power of the equipment are the most sensitive parameters for the extraction of phenolic acids from tangerine peel (*Citrus unshiu* Marc) using USN. The authors observed that using low temperatures (15 °C and 30 °C) and an increase in extraction time (10–40 min) and power (3.2 W to 30 W), increased the yield of the extracts. They also evidenced the effectiveness of UAE against CE (maceration), obtaining better results employing less extraction time. In the same line, Khan et al. [69] compared the extraction of polyphenols using UAE and CE of orange peel (*Citrus sinensis* L.). Although it is true that they did not determine the influential parameters in the results, they evidenced that the particle size of 2 cm^2^ favored a higher yield, recovering 38% and 41% more of naringenin and hesperidin, respectively, in contrast to the sample treated with CE. Recently, Jagannath and Biradar [62] concluded that UAE enhanced the bioactive compounds extraction and antioxidant activity of lemon peel (*Citrus limon* L.) extract against CE (Soxhlet extraction). Temperature and extraction time were the main parameters that influenced polyphenols, vitamin C content, and antioxidant activity of lemon peel extract.

Although the effectiveness of UAE to recover bioactive compounds against CE is evidenced, some studies reported different results. For instance, Garrido et al. [74], obtained a higher citrus seed oil yield, TPC, fatty acid content and antioxidant activity in samples from the Atacama desert treated by Soxhlet compared to UAE methods. In general, orange seed oil obtained by the Soxhlet method showed better results, however TPC in lime seed oil treated by USN was higher.

In the case of polyphenols extraction from sweet orange peels Nishad, Saha and Kaur [72] compared UAE and Enzyme Assisted Extraction (EAE) treatments. They observed that EAE enhanced the yield of the phenolic group, but UAE recovered a higher TPC (1590 mg GAE/100 g) and TF (104 mg QE/100 g) concentration compared to CE. The same range of TF was obtained by Van Hung et al. [74] in samples of grapefruit peel treated with UAE (TF = 190 mg RE/100 g). However, the simultaneous application of enzyme and UAE (EA-UAE) enhanced the yield of TF and TPC, compared to samples treated with UAE only. Also, EA-UAE performed the highest extraction yield of hesperidin and naringin from grapefruits peels. On the other hand, the range of hesperidin content extract by UAE from grapefruit peel (0.62–1.09 mg/g) samples is consistent with the hesperidin content (0.71 mg/g) reported by Wu et al. [58] from grapefruit peel treated by an ultrasound bath. However, the highest concentration of phenolic compounds, extracted from grapefruit peel (*Citrus paradisi* L.) by UAE was published by Nishad et al. [59]. They obtained 2116.71 mg GAE/100 g of TPC and 276.53 mg QE/100 g of TF and observed that TPC was affected (*p* < 0.05) mainly by amplitude, simple-solvent ratio, and their interaction.

Londoño-Londoño et al. [73] studied the effect of water content in sample and extraction time applied in UAE, on the polyphenols content of flavonoid fraction of tahiti lime (*Citrus latifolia*) peel, sweet orange (*Citrus sinensis*) peel and oneco mandarin (*Citrus reticulata*) peel. In this case, the highest yield was obtained with dry material and 30 min of extraction, also the statistical analysis showed that the water content influenced (*p* < 0.01) the TPC of the flavonoid fraction of the samples.

A short extraction time (15.05 min) was used by Dahmoune et al. [61] to extract phenolic compounds from *Citrus limon* peel with USN. They conducted an individual study of each parameter used. They reported 1522 mgGAE/100 g of TPC in lemon peel employing ethanol 63.93% as solvent. A lower concentration of TPC (1357 mgGAE/100 g) from orange peel was obtained a year later by Dahmoune et al. [70], who published a study where the concentration of the solvent and the amplitude of the equipment had a significant influence (*p* < 0.05) on the TPC. In this context, phenolic compounds in mandarin (*Citrus reticulata* Blanco cv. Sainampueng) and lime (*Citrus aurantifolia*) peels were compared by Singanusong et al. [64]. They addressed a similar study than Dahmoune et al. [70] but recovered a higher concentration of TPC (3083.61 mg GAE/100 g), TF (2539.82 mg QE/100 g), and hesperidin (1374.20 mg/100 g) in mandarin peel. However, the highest concentration of naringin was obtained in lemon peel (53.39 mg/100 g). This is explained by the polar affinity of the solvent and the extracted compounds and the differences between the varieties studied [61,70,71].

Mandarin and lemon peels were also analyzed by Saini, Panesar, and Bera [68]. They compared the TPC and TF of kinnow mandarin (*Citrus reticulata*) and sweet lemon “mousambi” (*Citrus limetta*) peel extracted by UAE treatment. In the study by Singanusong et al. [59], mandarin peel showed the higher concentrations of TPC (2800 mg GAE/100 g) and TF (440 mg catequin equivalent/100 g) against lime peel.

Subsequently, in 2017, Safdar et al. [65] compared the effectiveness of UAE against CE in the extraction of polyphenols (*Citrus reticulata* L.) from mandarin peels, with different solvents at different concentrations. They obtained the highest concentration of TPC in UAE samples (3248 mg GAE/100 g) using methanol 80% as solvent. They also showed that the major compound in mandarin peel was hesperidin (929 mg/100 g), using ethanol 80% as solvent.

Nipornram et al. [66] also used UAE to extract phenolic compounds from mandarin peel (*Citrus reticulata* Blanco cv. Sainampueng). They observed that the interaction between extraction temperature and potency significantly influenced (*p* < 0.05) the extraction yield and TPC in mandarin peel. An extraction yield of 26.49%, 15256.64 mg GAE/100 g of TPC, and a hesperidin content of 6444.84 mg/100 g were obtained. These are the highest values found for phenolic compounds extracted from mandarin peel. However, this was possible employing also an organic solvent (acetone 80%). In contrast, deep eutectic solvents (DES) would be the best option in terms of sustainable efficient extraction [86]. For instance, in a similar study, Xu et al. [67] showed that 80% Choline chloride: Levulinic acid: N-methyl urea (ChCh–LeA–MU) allowed recovering the highest TF content (6582 mg/100 g) from mandarin peel treated by UAE. In addition, a shorter extraction time (25′) was required, compared to the study by Nipornram et al. [66].

On the other hand, a low concentration of TPC was obtained by Boukroufa et al. [71] from orange peel (50.02 mg GAE/100 g) using residual water (from a previous extraction with Microwave hydrodiffusion and gravity) as solvent during the ultrasound treatment. The particle size selected was 2 cm^2^ according to the study by Khan et al. [69] for orange peel samples prior to treatment.

The peel of citrus fruits is rich in pectins, a complex carbohydrate, chemically structured as a galacturonic acid polymer with a variable number of methyl ester groups, and the main cell wall component of citrus waste [87]. This compound is widely extracted with conventional methods for its use as a byproduct. Several studies suggest good extraction results with the use of an unconventional methodology such as UAE. For example, Bagherian et al. [76] used USN treatment for the extraction of skin pectins (albedo) from grapefruits. With an extraction time of 25 min and using acidified water at a pH of 1.5 with 0.1 N HCl as extracting agent, they obtained a 17.92% yield of pectins.

Meanwhile, Wang et al. [77] investigated the extraction of pectins from grapefruit peel by ultrasound assisted heating extraction (UAHE) compared with conventional heating extraction (CHE). Deionized water adjusted by 0.5 M HCl until pH 1.5 was used as extraction solvent. The pectin yield of grapefruit peel treated by UAHE was 16.34% higher than pectin yield extracted by CHE. The authors observed that power intensity and extraction temperature influenced the pectin yield (*p* < 0.05), as well as and the interaction between power intensity with sonication time, and extraction temperature with sonication time. In addition, the degree of esterification, polyphenols and flavonoids content of the pectins extracted by UAHE were 65.52%, 4.21 µg GAE/mg, and 1.76 µg RE/mg, respectively. Subsequently, Hosseini et al. [78] showed that extraction power, time and pH significantly affected (*p* < 0.01) the pectin yield from bitter orange (*Citrus aurantium* L.) peel obtained by UAE.

Since the advantages of USN treatment against conventional methods and the potential use of green solvents are evidenced, industrial scale research of the use of UAE with citrus residues should to be studied in more detail. Also, the simultaneous application of ultrasound treatment with another type of novel technology is a promising method to extract high added value compounds from citrus waste. For instance, Liew et al. [88] obtained the highest (36.33%) pectin yield from grapefruit peel employing ultrasound and microwave extraction combined.

### 2.2. Microwave Assisted Extraction

The microwave technique is characterized by using electromagnetic waves at a frequency of 2.45 GHz, but according to the literature it ranges from 0.3 GHz to 300 GHz [89,90]. In microwave assisted extraction (MAE), the waves interact with polar molecular compounds such as water, causing damage to citrus residues due to the increase in heat and pressure inside the cell walls. The porosity caused favoring the transfer of waste molecules and as a consequence, an improvement in the yield of the extracts [90,91] without affecting the compounds. Low extraction time and energy consumption are the main advantages.

The short time required for its application, reduced the use of solvents and enhanced its effectiveness compared to other extraction methods (conventional and non-conventional) [92]. For that reason, the MAE technique has been widely used for the extraction of bioactive compounds (mainly polyphenols and essential oils) and pectins from citrus byproducts (Table 2). Likewise, in several studies, RSM is applied to optimize the parameters used in this technology [93,94,95].

The main parameters that influence the efficiency of the microwave extraction are power; frequency (microwave energy); extraction time; temperature, moisture content, type, and concentration of the solvent; and the sample–solvent ratio employed; the number of extraction cycles; and the particles size (samples) [91]. On the other hand, Wang and Weller [90] stated that the most critical parameter is the solvent, since greater polarity improves energy absorption and increases the yield of the molecules to be extracted. However, it is advisable to use mixtures of solvents with water, as the polarity is favored, and its loss due to dissipation is reduced, allowing a reduction in the use of organic solvents. Thus, Hayat et al. [93] recovered 116.42 mg/100 g of free phenolic acids from mandarin peel (*Citrus reticulata* Blanco cv Kinnow) treated by MAE using 66% aqueous methanol as solvent. The content of free phenolic acids obtained with MAE was higher than that obtained by UAE (114.23 mg/100 g) and CE (63.24 mg/100 g).

Higher concentrations of free phenolic acids (117.9 to 132.9 mg/1100 g were reported a year later by Hayat et al. [94], who employed a higher methanol concentration (80%) in the treatment with MAE to extract phenolic compounds from mandarin peel (*Citrus reticulata* Blanco cv Kinnow). A high concentration of the total flavonoids sum (637.59 mg/100 g) was also obtained. These studies show an increase in the extraction yield of phenolic compounds in free form by means of MAE increasing the antioxidant activity of mandarin peel and favoring the effectiveness of the method

Inoue et al. [95] focused on optimizing the parameters to be used in the treatment of MAE for the extraction of flavonoids from mandarin peel using 70% aqueous ethanol. The hesperidin yield obtained was 27 higher (5860 mg/100 g) by CE (217.04 mg/100 g). Also, MAE evidenced a higher concentration of narirutin (1310 mg/100 g) compared to CE (524 mg/100 g). A similar study was conducted by Ahmad and Langrish [97], who used microwaves to prepare an aqueous extract of tangerine peel (*Citrus reticulata*) to recover phenolic compounds. They observed that the increase in temperature enhanced the TPC concentration (2320 mg GAE/100 g) in a short period of time (3 min). Aqueous extracts made by MAE were used by Caputo et al. [106]. They treated peel powder of citrons, sweet oranges and lemons at different temperatures (80–100 °C) and extraction time values (8–20 min), obtaining an extraction yield between 18–21.5%. However, citrus peel origin and extract type influenced the results. Thus, the extraction yield of lemon peel powder is favored by a short extraction time.

In contrast, Dahmoune et al. [61] observed in their study that the TPC of citrus lemon peel was affected (*p* < 0.01) by the sample-solvent ratio and, in turn, by the concentration of solvent (ethanol) and the extraction power. They found that the increase of ethanol concentration up to 50% and the power of 400 W increased the TPC yield (1330 mg and 1368 mg GAE/100 g, respectively). Also, the solid–solvent ratio of 1:25 (g/mL) allows the extraction of a higher content of TPC (1472 mg GAE/100 g).

Nayak et al. [97] also evaluated the effect of the solvent type, power, solid–solvent (mg/mL) ratio, and extraction time during MAE to obtain orange peel phenolic compounds (*Citrus sinensis*). They found that the type of solvent influenced the extraction of TPC obtaining greater extraction with 50% acetone compared to water, ethanol 50% and methanol 50%. Likewise, a short extraction time (90–150 s) favored the TPC content. The research group evidenced that compared to accelerated solvent extraction and conventional extraction, MAE significantly enhanced (*p* < 0.01) the extraction of TPC and some phenolic acids such as caffeic and ferulic acids. Microwave treatment was evidenced as a fast and reliable method for the extraction of phenolic compounds from citrus residues with lower solvents expenditure [61,96,97].

This technology has also been used to extract essential oils (EOs) from citrus residues. These secondary metabolites secreted by plants are used as a defense mechanism against microorganisms since they inhibit fermentation processes and are located in small vesicles of the flavedo or citrus exocarp [107]. The peel of citrus fruits contains more than 200 EOs compounds, both volatile and non-volatile [108] and the main compound of the citrus EOs composition is limonene (30–70% depending on the variety) [109].

Ferhat et al. [101] indicated that microwave accelerated distillation (MAD) is the best option to extract EOs from orange peel (*Citrus sinensis* L. Osbeck), obtaining a high limonene content (76.7%) using a 30 min treatment at 100 °C without solvent. Likewise, the yield of the essential oil extract under these conditions by MAD is greater (0.42%) than that obtained by hydro-distillation (HD) (0.39%). Likewise, the fraction of oxygenated monoterpenes was higher in the extract obtained by MAD than HD. In this line, MAD could be a potential option to extract EOs from citrus waste in terms of saving.

A similar study was conducted by Farhat et al. [102] to determine the optimal parameters to be used in microwave steam distillation (MSD) equipment. They obtained higher EOs yield from orange peel (*Citrus sinensis* L. Osbeck) with the microwave treatment (1.54%) than CE (steam distillation) (1.51%). The major essential oil in samples extracted with microwaves was limonene (94.88%), followed by β-myrcene and linalool (1.59% and 0.29%, respectively). The data obtained suggest that the use of microwaves enhanced the EOs extraction from orange peel with a lower energy and time expenditure, compared to conventional steam distillation.

Bustamante et al. [100] compared the essential oils yield of orange peel extracts obtained by MAE-HD and conventional HD. A 1.8% yield of EOs was obtained by MAE, which was slightly higher than the yield obtained by HD. Due to this, a significant reduction in energy consumption was observed in MAE-HD compared to conventional HD (0.5 kWh and 3.2 kWh, respectively). The major compound was limonene (97.38%), and other compounds of interest, such as γ-terpinen and trans-a-bergamoten, could also be detected.

In relation to energy consumption, Gonzales-Rivera et al. [103] conducted a study to evaluate de efficiency of microwave treatment in different variations, in the EOs recovery from orange peel. They evidenced that solventless–microwave assisted extraction (S-MAE) is the most promising approach to reduce the energy consumption since it was around 27 times lower than the energy required by conventional HD. A 95.2% limonene yield was obtained from orange peel by S-MAE.

In other study carried out to obtain EOs of orange peel (*Citrus sinensis* var. Valencia), it was shown that the particle size, moisture content and interaction between both significantly affected (*p* < 0.05) the EOs yield. It was observed that smaller particle size (40 µm) and lower moisture content (10%) improved the extraction of EOs (2.73%). They also noted that a high microwave power required less extraction time to recover EOs from orange peel [99]. A lower extraction yield was obtained by Auta et al. [104], i.e., 3.7% and 2.0% of EOs yield from orange and lemon peel.

On the other hand, different types of EO constituents were obtained by MAE treatment from bergamot (*Citrus bergamia* Risso) by-product. In this case, Di Donna et al. [98], recovered brutieridin and melitidin from bergamot albedo oil employing a short extraction time. Also, they observed that the amount of the extracted species decreased with increasing extraction time (1 to 5 min) and solvent temperature (48 °C to 82 °C).

Regarding the extraction of pectins, Bagherian et al. [76] obtained a pectin yield of 27.81% from grapefruit peels treated with MAE and using as parameters 900 W of power and 6 min of extraction. Comparing UAE (17.92%) and CE (19.16%), MAE was evidenced as the most efficient method to extract pectin from citrus peel. Also, they showed that increasing power, a shorter extraction time is required to enhance the pectin yield.

A similar study was conducted by Maran et al. [87] to optimize the parameters to be used with MAE treatment for the extraction of orange pectins (*Citrus reticulata*). They observed that the yield of pectins increased with increasing equipment power in a short irradiation time. 19.19% of pectin yield was obtained employing 422 W in a shorter irradiation time (169 s). With similar parameters, Boukroufa et al. [71] used residual water extract pectins from orange peel (*Citrus sinensis* L. Osbeck) during MAE treatment. They observed, just like Bagherian et al. [76] and Maran et al. [87], that with a higher power and lower extraction time, the pectin yield is enhanced. They showed the highest yield (24.2%) at 500 W and 3 min of extraction compared to CE (18.32%) during 2 h of treatment.

The highest pectin yield was obtained by Hosseini et al. [105], who conducted a study to extract pectins from sour orange peel (*Citrus aurantium* L.) using microwave treatment. They obtained 29.1% of pectin yield during 3 min of irradiation and 700 W of potency.

MAE is the non-conventional method, which required lowers extraction times to obtain notable yields of bioactive compounds and pectins, commonly higher than yields obtained by CE. This short extraction time is reached with less energy consumption, and as a consequence, shows the efficiency of MAE as a potential alternative at industrial scale to extract high biological value compounds from citrus byproducts.

### 2.3. Supercritical Fluid Extraction

Supercritical fluid extraction (SFE) is a procedure that combines fluids at high temperatures and pressures with values close to the critical points. These fluids have a high diffusivity and low density, viscosity, and surface tension compared to organic solvents [110]. Due to these characteristics, porous solid materials can enter more effectively than with solvents liquids, resulting in a faster mass transfer and extraction than with solvent extraction methods [111]. This technique is designed to replace traditional sample preparation techniques that include several steps and that use large amounts of organic solvents. The effectiveness of SFE also arises, in part, from changes in the solvation power with changes in density and, therefore, the temperature and fluid pressure around the critical point [112].

Carbon dioxide (CO_2_) is the supercritical fluid most used in this method, often for the extraction of bioactive compounds in a safe and environmentally friendly way and with the advantage for minimally affecting the extracted compounds [113]. Different parameters such as temperature (critical at 31 °C), pressure (74 bar), SFE-CO_2_, and presence of co-solvents such as ethanol and methanol, could influence the efficiency of this method [42]. The solvation power of the SFE-CO_2_ fluid can be increased or decreased by manipulating pressure and/or temperature, resulting in high selectivity and separation of dissolved solutes [113]. In citrus fruit residues, this technique is applied for the extraction of essential oils; however, the extraction of other compounds has been described (Table 3).

The presence of solvents is important to improve the efficiency of extraction by increasing solubility of the solute [42]. On the other hand, due to the a-polar affinity of SFE-CO_2_, this method limits its action to polar compounds such as polyphenol. Therefore, the use of co-solvents such as methanol, ethanol and water are required to improve the polarity of SFE-CO_2_ and allow the extraction of polar compounds [110].

In this line, Tsitsagi et al. [114] used SFE-CO_2_ to extract different bioactive compounds from mandarin peel (*Citrus unshiu*). An extraction yield of 0.85% was obtained, and limonene was the majority compound in the samples. With the use of CO_2_-acetone (7%) between 0.04 mg and 0.39 mg of β-carotene/100 g peel were extracted and with CO2-methanol (7%) a yield between 1.6% and 1.8% of hesperidin was obtained. Trabelsi et al. [115] applied the SFE-CO_2_ technology as a treatment for the extraction of bioactive compounds from bitter orange peel (*Citrus aurantium* amara). They used ethanol 3% as a co-solvent. At 170 bar, 53 min of static time and CO_2_ flow rate 2.87 kg/h, a 1.07% of extraction yield was obtained. Osthol was the major compound (47%) extracted in orange peel samples. They also found squalene, hexadecane, and esters of fatty acids. The statistical analysis indicated that the pressure, the flow and the interaction between the pressure and the static time, and between pressure and flow, significantly affected (*p* < 0.05) the extraction yields.

Although limonene is the major EO extracted from citrus waste samples treated by SFE-CO_2_, in a study conducted by Menichini et al. [116], the major constituent of citron (*Citrus medica*) peel oil was citropten (84.5%). In contrast, traces of limonene were identified.

Omar et al. [75] used SFE to obtain phenolic compounds and EOs from mandarin, orange, lemon and grapefruit peel. For the extraction of phenolic compounds, they used a pressure of 160, flow of 1 mL/min, 35 °C and 40% aqueous ethanol, obtaining a higher concentration of TPC in grapefruit skin (0.67 mg GAE/100 g), followed by lemon peel, orange, and tangerine (0.66, 0.45, and 0.38 mg GAE/100 g, respectively). In relation to EOs, they found that temperature and flow rate were the parameters that most influenced (*p* < 0.05) the recovery of these compounds, especially limonene, β-pinene and γ-terpinen. Lopresto et al. [117] demonstrated that the maturing stage influenced the oil yield and D-limonene extraction from lemon (*Citrus limon* L.) peel. The highest content of essential oil and D-limonene in essential oil was reached in samples collected in December. They also observed that particle size plays an important role in the SFE treatment.

### 2.4. Pressurized Water Extraction

Pressurized water extraction (PWE) is also known as subcritical water extraction (SWE), and its mechanism of action begins with desorption of solutes from various active sites in the samples to be analyze, under conditions of high pressure and temperature. This allows the diffusion of the extraction fluid in the matrix; then, the compounds can be dragged into the extraction fluid and finally analyzed chromatographically [118]. The extraction power of water is based on that at high pressure and temperature levels, the affinity of a-polar compounds increases. This is similar to organic solvents such as methanol and ethanol [119]. Teo et al. [118] also indicated that analytes extracted by this method are safe for further analysis, processing and human consumption.

PWE is a method based on the use of water as a solvent at high pressure, generally between 4 MPa and 20 MPa, requiring less extraction time and low or zero expenditure of organic solvents. The optimization of the parameters according to the type of compound and sample to be analyzed is advisable. According to Cravotto and Cintas [120], the main parameters to consider in this method are pressure, flow rate, temperature and extraction time, and in other studies, factors such as particle size and static time per cycle were also considered influential [121].

In this line, Cheigh, Chung, and Chung [122] showed that temperature and extraction time are the main factors that influenced (*p* < 0.05) the recovery of hesperidin and naritunin from mandarin peel (*Citrus unshiu*). A temperature of 160 °C, 10 min of extraction and pressure of 100 atm were used to obtain 7200 mg/100 g and 11,700 mg/100 g of hesperidin and narirutin, respectively. They also indicated that subcritical water could be an excellent alternative to organic solvents to extract nonpolar citric flavanones, in terms of efficiency and respect for the environment.

Lachos-Perez et al. [123] used SWE to extract flavanones from defatted orange peel. They showed that when the temperature increased, the extraction yields increased, and the interaction between temperature and flow rate influenced (*p* < 0.05) the global yield. The highest extraction yield (10.63%) was obtained at 150 °C with a water flow rate of 10 mL/min. With these conditions, 20 mg/g and 2.33 mg/g of hesperidin and naritunin were obtained, respectively, and 31.70 mg GAE/g of TPC was extracted from defatted orange peel. Also, the pectin yield was estimated at 18.53%, according to Wang, Chen, and Lü [124], who obtained 21.95% of pectin yield from mandarin peel. Their statistical analysis indicated that temperature significantly affected pectin yield as well as galacturonic acid content.

In other studies, water is replaced by different types of solvents. This technique is known as pressurized liquids extraction (PLE). PLE has emerged as a powerful method to recover polar molecules from fruit byproducts, such as phenolic compounds and EOs, using environmentally friendly solvents, like CO_2_ and ethanol [125]. For instance, Barrales et al. [125] conducted a study with orange peel from a juice factory, treated with PLE to determine the content of phenolic compounds among other compounds. They worked with two samples (lots 1 and 2). Lot 1 was previously treated with SFE-CO_2_ to extract EOs. Subsequently, both samples (lots 1 and 2) were treated with PLE using ethanol 75% as solvent. The results showed that in lot 1, the major oils were α-terpinol (76%) and D-limonene (18.80%). TPC was mostly present in lot 2 with a total of 1590 mg GAE/100 g of peel. Hesperidin was the major flavonoid in both samples (5800 mg/100 g and 5400 mg/100 g peel in lot 1 and 2, respectively).

### 2.5. Pulsed Electric Field

The pulsed electric field (PEF) applied in the extraction of specific bioactive compounds is a non-conventional technology that does not affect the quality of the extracts. This technique is based on submitting a sample during a short time (1–2500 µs) to a strong electrical field (10–80 kv/cm) between two electrodes; the stress generated favors the pore formation in the membranes [50,126,127]. A phenomenon known as membrane electroporation [128] increases the transfer between the solvent and the compounds contained in the sample from one area to another. This phenomenon is influenced by the strength of the electric field; the number and duration of pulses used [50]; the treatment time (t PEF = number of pulses × pulse duration); the pulse waveform, conductivity, pH, and ionic strength of the medium; and the shape and size of the sample [129].

Some studies detail the application of PEF on citrus fruit residues due to the shorter treatment time and lower energy expenditure, favoring the quality of the extracts. Luengo, Álvarez, and Raso [130] determined that the use of PEF improved the extraction under pressure of orange peel polyphenols, by obtaining a higher quality extract and antioxidant capacity, reducing extraction times and even without using organic solvents. Due to the effect of permeabilization of the PEF on the orange peel cell membrane, facilitating the release of the phenolic compounds inside the cells and their consequent extraction. Also, with 5 kV/cm, the naringin and hesperidin content increased from 1–3.1 mg/100 g and 1.3–4.6 mg/100 g of orange peel, respectively.

Recently, El Kantar et al. [131] compared the total polyphenol content in juice and peel (flavedo/albedo) of orange, grapefruit, and lemon. A whole fruit sample was initially pretreated with PEF at 3 kV/cm in order to be compared with the non-pretreated samples. A conventional extraction with 50% ethanol was performed for 1 h in both samples, to be treated once more with PEF at 10 kV/cm. The research group observed an increase in the flavonoid content of orange, grapefruit and lemon pre-treated peels. Also 2200 mg GAE/100 g of TPC in orange peel treated with PE 10 kV/cm was obtained. In conclusion, the use of PEF improves the performance of total polyphenols and some flavonoids, due to the electroporation phenomenon, compared to untreated samples.

Some studies concerning the extraction of bioactive compounds by PWE, PLE, and PEF are summarized in Table 4.

### 2.6. High Voltage Electric Discharges

High voltage electric discharge (HVED) technology is considered a “green” method for the extraction of bioactive compounds. This method favors the extraction yield with the advantages of lower energy expenditure [132], requiring a short extraction time and a minimum increase in temperature during the treatment. HVED is based on the electrical rupture in the water by the action of the applied discharges, which allows the creation of an avalanche of electrodes that propagate in intense electric fields [133]. Defragmentation and damage to the cell membranes of the samples are generated by the action of shock waves, cavitation bubbles, and turbulence caused by the action of “electrical breakdown” due to electrical discharges and the consequent increase in pressure [132,133]. Boussetta and Vorobiev [132] suggest that the parameters to be considered in this technology are the treatment of energy input, distance between the electrodes, sample-solvent rate, temperature and extraction time, and solvent. The contents of high biological value compounds from citrus waste extracted by HVED are shown in Table 5.

Buniowska et al. [134] determined the content of bioactive compounds of an orange peel extract (*Navel Navelate*) obtained by HVED. The researchers used a voltage of 40kV with discharges per 10 µs, and applied two energy inputs, 55 and 364 kJ/kg. They observed that the increase of energy favored the bioaccessibility of carotenoids (82.5%), while the highest % of bioaccessibility of phenolic compounds (40.7%) was observed at 55 kJ/kg.

On the other hand, El Kantar et al. [135] used HVED as a pre-treatment before diffusion to extract phenolic compounds from grapefruit peel. At 10 min of diffusion in water, the TPC of grapefruit peel treated with DEAV increased from 1330 mg/100 g to 1880 mg/100 g of peel according to the energy increase from 0 kJ/kg to 218 kJ/kg, respectively. At the same time, the energy employed in the HVED treatment can be reduced if the subsequent extraction is conducted in aqueous glycerol, since the increase in glycerol concentration from 10% to 30%, increased the TPC from 1770 mg/100 g to 1930 mg/100 g dry peel.

A short extraction time and a low or zero solvents necessity are the main advantages of HVED in the context to obtain safe byproducts extractions.

### 2.7. High Hydrostatic Pressures

Initially studied as an alternative to pasteurization [84], high hydrostatic pressure (HHP) is one of the most researched non-thermal treatments used in liquid or solid products. This treatment is based on the application of high pressure (100–800 MPa, even up to 1000 MPa) evenly and quickly in the sample through a liquid phase, which is commonly water, improving the mass transfer rate, the solvent permeability in cells, and the diffusion of secondary metabolites [114,136]. It is characterized by not being an invasive technique, and its involvement in the inactivation and/or inhibition of microorganisms has also been investigated [137].

The application of high pressure affects the cell walls and hydrophobic bonds in the cell membrane of the citrus peel, favoring high solvent permeability and greater contact with the compounds during extraction in a short time [136]. Concentrations of some biological compounds extracted by HHP from citrus by-products are showed in Table 6. In a first study, Casquete et al. [138] determined the content of TPC and antioxidant capacity of lemon and orange peel, treated at 300 MPa and 500 MPa for 3 min and 5 min. They observed that the choice of parameters is dependent on the matrix to be used, i.e., 300MPa for 10 min for orange peel and 500 MPa for 3 min for lemon peel. Under these conditions, 136.85 mg and 344.53 mg GAE/100 g of TPC and 136.85 mg and 149.41 mg *trolox*/100 g (antioxidant capacity) were obtained in orange and lemon peel, respectively.

A similar study was conducted a year later by Casquete et al. [19], who compared the application of HHP at different variations in samples of lemon, lime, tangerine, and sweet orange peel. They obtained higher values of total polyphenols in the samples treated at 300 MPa for 3 min, this also correlated with a higher antioxidant capacity compared to the other data (except in the case of tangerine skin that indicated a higher antioxidant capacity at 500 MPa/10 min). They also indicated that the equilibrium of the pressure between the inside and outside of the sample cells occurred in a very short time, and the extraction of phenolic compounds reached the highest value very quickly, explaining a higher content (*p* < 0.01) of TPC and antioxidant capacity at 3 min of the treatment. The application of this treatment to citrus fruit residues was also evaluated to improve its dietary fiber potential [139], obtaining pectins [140], and inactivation of microorganisms [141].

## 3. Conclusions

The current interest to reduce industrial and food waste is in agreement with the sustainable objectives of the United Nations by 2030. In the last decade, the attention of researchers to obtain bioactive compounds from food waste such as citrus byproducts, using environmentally friendly technologies has increased. These compounds can be reused in the food, pharmaceutical, and cosmetic industry with promising results. It is known that conventional methods allow the recovery of bioactive compounds and pectin from citrus byproducts. However, a high expenditure of time and energy, due to the use of organic solvents, have made conventional methods an unfavorable option. In contrast, the replacement of non-conventional methods for the extraction of high biological value compounds from citrus by-products has shown a large number of advantages in a green extraction context. An optimization study is performed by most of the researchers who apply non-conventional methods, since some parameters can influence the results, according to the characteristics of the selected technologies and samples. The type of waste and the molecules to be extracted, are the main variables to be considered in the selection of the most appropriate technology. However, more bioaccessibility, bioavailability, and validation studies of high biological value compounds from citrus waste are needed. Also, an industrial scale application of green technologies with citrus byproducts has not been performed yet. Despite the equipment cost, the sustainable characteristics of non-conventional methods suggest potential results in this scenario. Moreover, recently, some researchers also focused on the use of alternative solvents such as DES for extracting bioactive compounds from food byproducts. Their coaxial application with non-conventional methods in the extraction of citrus waste bioactive compounds is a promising research line.

## Figures and Tables

**Table 1:** Content of high-value biological compounds from citrus wastes extracted with ultrasound assisted extraction (UAE).

Compound	Sample	Extraction Conditions	Solvent	Results	References
Carotenoids	Mandarin peel (*Citrus spp.* N/A)	66.7% amplitude 25 °C 544.59 W/cm^2^ ratio 3:30 (g/mL) 20–120 min	Ethanol	The use of ultrasound increases the extraction performance of all-trans-β-carotene compared to CE	[53]
	Kinnow mandarin peel (*Citrus reticulata*)	32.8% amplitude 43.14 °C 33.71 min ratio 1:6.16 (g/mL)	N/A	Maximum lutein yield 2.97 mg/100 g	[54]
	Orange peel (*Citrus spp.* N/A)	208 W/cm^2^ 20 °C 5 min	Limonene (previous extraction)	Total carotenoids: 1.13 mg β-carotene/100 mL of extraction 40% higher yield than conventional extraction	[55]
	Orange peel (*Citrus sinensis*)	400 W 30 min 40 °C ratio1:10 (g/mL)	Ethanol 50%	Total carotenoids: 0.63 mg β-carotene/100 g	[56]
	Orange peel (*Citrus spp.* N/A)	200 W 5 min ratio 1:3 (g/mL) 80% amplitude	ILs[BMIM][CL]	Total carotenoids: 3.20 β-carotene/100 g	[57]
Phenolic compounds	Grapefruit peel (*Citrus spp.* N/A)	2 h ratio 1:10	Ethanol 99.7%	Naringin: 1400 mg/100 g Hesperidin: 7.14 mg/100 g	[58]
	Grapefruit peel (*Citrus paradise* L.)	71.11% amplitude 33.12 min ratio 1:39.63	Ethanol 70%	TPC: 2116.71 mg GAE/100 g TF: 276.53 mg GAE/100 g Naringin: 4203 mg/100 g Quercitin: 12.097 mg/100 g	[59]
	Grapefruit peel (*Citrus grandis limonia* Osbeck species)	40 kHz 60 min room temperature ratio 1:40 (g/mL)	Water	TPC: 408–687 mg GAE/100 g TF: 93–190 mg RE/100 g Naringin: 13–49 mg/100 g Hesperidin: 62–109 mg/100 g	[60]
	Lemon peel *(Citrus limon*)	77.79% amplitude 15.05 min	Ethanol 63.93%	TPC: 1522 mg GAE/100 g	[61]
	Lemon peel (*Citrus limon* L.)	200 W 80% duty cicle 60 min 48 °C ratio 1:6 (g/mL)	Methanol 80%	TPC: 67.17 mg GAE/100 g TF: 4.52 mg catequin equivalent/100 g	[62]
	Mandarin peel (*Citrus unshiu marc*)	60 kHz 3.2–30 W 15–40 °C 10–60 min	Methanol 80%	High yield at 15 °C and 60 min, compared to maceration and ultrasound at 40 °C and 60 min	[63]
	Mandarin peel (*Citrus reticulata* Blanco cv) and lime peel (*Citrus aurantifolia*)	50.93 W	Acetone 80%	Mandarin TPC: 3083.61 mg GAE/100 g TF: 2539.82 mg QE/100 g Hesperidin: 1374 mg/100 g Lime Naringin: 53.39 mg/100 g	[64]
	Mandarin peel (*Citrus spp.* N/A)	35 kHz 45 °C ratio 1:20 (g/mL) 60 min	Methanol 80%	TPC: 3248 mg GAE/100 g Hesperidin: 5.21 mg/100 g	[65]
	Mandarin peel (*Citrus reticulata* Blanco cv. Sainampueng)	56.71 W 48 °C 40 min	Acetone 80%	Extraction yield 26,52%, 1.77 times higher than CE TPC: 15256.64 mg GAE/100 g Hesperidin: 6444.84 mg/100 g	[66]
	Mandarin peel (*Citrus unshiu*)	200 W 25 min 50 °C	ChCl-Lea-MU 80%	TF: 6582 mg/100 g polymethoxylated flavonoids (PMFs): 1875 mg/100 g glycosides of flavonoids (GoFs): 4707 mg/100 g	[67]
	Kinnow mandarin peel (*Citrus reticulata*) and sweet lemon “mousambi” peel (*Citrus limetta*)	ratio 1:3 (g/mL) Other parameters N/A	Acetone 100%	Mandarin Extraction yield 5.85% TPC: 2800 mg GAE/100 g TF: 440 mg GAE/100 g Sweet lemon Extraction yield 12.95% TPC: 2199 mg GAE/100 g TF: 207 mg GAE/100 g	[68]
	Orange peel (*Citrus sinensis*)	150 W 40 °C 30 min ratio 0.25:1 (g/mL) 2 cm^2^ particle size	Ethanol 80%	Extraction yield TFC: 275.8 mg GAE/100 g Extraction yield: 10.9% higher compared CE Naringin: 70.3 mg/100 g Hesperidin: 205.2 mg/100 g	[69]
	Orange peel (*Citrus spp.* N/A)	8.33 min 65.94% amplitude	Acetone 75.79%	TPC: 1357 mg GAE/100 g	[70]
	Orange peel (*Citrus spp.* N/A)	0.956 W/cm^2^ 59.83 °C ratio 1:10 (g/mL) 30 min 2cm^2^ particle size	Water (previous extraction)	TPC: 50.02 mg GA/100 g	[71]
	Sweet orange peel (*Citrus sinensis* cv. Malta)	70.89% amplitude 35 min room temperature ratio 1:40 (g/mL)	Etanol 70%	TPC: 1590 mg GAE/100 g TF: 104 mg QE/100 g Naringin: 1.10 g/100 mL of extract	[72]
	Lime, orange, mandarin peel (*Citrus spp.* N/A)	60 kHz 30 min 40 °C ratio 1:10 (g/mL)	Water	TPC 7480 mg GAE/100 g lime 6636 mg GAE/100 g orange 5868 mg GAE/100 g mandarin	[73]
	Lime and orange seed oil (*Citrus spp. N/A*)	100 W 90 min 25 °C	n-Hexane	Lime seeds TPC: 65.3 mg GAE/100 g oil Orange seeds: TPC: 68.2 mg GAE/100 g oil	[74]
	Citrus fruits peel (*Citrus spp.* N/A)	5 min 30% amplitude Ciclo 5 s^−1^	Ethanol	TPC: 1259 mg GAE/100 g (orange) 1248 mg GAE/100 g (grapefruit) 1812 mg GAE/100 g (lemon) 793 mg GAE/100 g (mandarin)	[75]
Essential oils	Mandarin, orange, grapefruit and lemon peel (*Citrus spp.* N/A)	5 min 30% amplitude ratio 0.25: 15 (g/mL) cycles 5 sec^−1^	Ethanol 100%	Limonene: 3010 mg/100 g (mandarin) 1360 mg/100 g (grapefruit) 1280 mg/100 g (orange) 140 mg/100 g (lemon)	[75]
	Lime and orange seed oil (*Citrus spp.* N/A)	100 W 90 min 25 °C	n-Hexane	Lime seeds Oil yield: 22.07% Linoleic acid: 34.07% α-linoleic: 11.45% Orange seeds: Oil yield 22.84% Linoleic acid: 34.07% α-linoleic: 11.45%	[74]
Pectins	Grapefruit peel (*Citrus spp.* N/A)	25 min 70 °C	Acidified water	Pectin yield: 17.92%	[76]
	Grapefruit peel (*Citrus spp.* N/A)	12.56 W/cm^2^ 28 min 67.7 °C ratio 3:150 (g/mL)	Acidified water	Pectin yield: 27.34% Degree of Esterification: 65.52%	[77]
	Sour Orange peel (*citrus aurantium* L.)	150 W 10 min pH 1.5 ratio 1:20 (g/mL) <30 °C	Acidified water (cítric acid)	Pectin yield: 28.07% Galacturonic acid: 65.3% Sugars: 0.4% (74% galactose) TPC: 3995 mg GAE/100 g of pectin	[78]

Abbreviations: conventional extraction, (CE); not available, (N/A); ionic liquids, (ILs); chlorinated 1-Butyl-3-methylimidazolium, (BMIM-Cl); total phenolic compounds, (TPC); galic acid equivalent, (GAE); total flavonoids, (TF); quercitin equivalent, (QE); rutin equivalent, (RE).

**Table 2:** Content of high biological value compounds from citrus wastes extracted with microwave assisted extraction.

Compound	Sample	Extraction Conditions	Solvent	Results	References
Phenolic compounds	Lemon peel (*Citrus limon*)	Ratio 1:28 (g/mL) 400 W 120 s	Ethanol 48%	TPC: 1574 mg GAE/100 g	[61]
	Mandarin peel (*Citrus reticulata* Blanc*o* cv. Kinnow)	152 W 49 s	Methanol 66%	Free phenolic compounds: 1162.8 mg/100 g Free phenolic acids: 114.23 mg/100 g	[93]
	Mandarin peel (*Citrus reticulata* Blanco cv. Kinnow)	250 W 10 min	Methanol 80%	TFs: 637.59 mg QE/100 g Total phenolic acids fraction: 131.57 mg/100 g	[94]
	Mandarin peel (*Citrus inshiu*)	Ratio 2:20 (g/mL) 7 min 140 °C	Ethanol 70%	Hesperidin: 5860 mg/100 g Narirutin: 1310 mg/100 g	[95]
	Mandarin peel (*Citrus spp.* N/A)	Ratio 1:2 (g/mL) 400 W 3 min 135 °C	Deionized water	TPC: 2320 mg GAE/100 g	[96]
	Orange peel (*Citrus sinensis*)	Ratio 1:25 (g/mL) 500 W 122 s	Acetone 51%	TPC: 1220 mg GAE/100 g Cafeic acid: 81.59 mg/100 g Ferulic acid: 145.5 mg/100 g	[97]
Essential oils	Bergamot albedo (*Citrus bergamia* Risso)	500 W 1–5 min <48 °C Ratio 3:20 (g/mL)	Water	Brutieridin Melitidin	[98]
	Mandarin peel (*Citrus sinensis* var. Valencia)	Particle size 40 µm 540 W 10–20 min	N/A	EOs yield: 2.73%	[99]
	Mandarin peel (*Citrus sinensis* var. Navel Navelate)	Ratio 1:1,5 (g/mL) *Part 1:* −785 W −5 min *Part 2:* −250 W −15 min	Water	EOs yield: 1.8% Monoterpene hydrocarbons: 99.34% Limonene: 97.38%	[100]
	Orange peel (*Citrus sinensis* L. Osbeck)	30 min 100 °C	N/A	EOs yield: 0.42% Limonene: 76.7% Oxygenated monoterpenes: 7.0%	[101]
	Orange peel (*Citrus sinensis* L. Osbeck)	200 W 12 min	N/A	EOs yield: 1.54% Monoterpene hydrocarbons: 98.23% Limonene: 94.88% Oxygenated monoterpenes: 0.43%	[102]
	Orange peel (*Citrus spp.* N/A)	250 W 5 min	N/A	EOs yield: 1.16% Limonene: 95.2 Valencene: 0.2%	[103]
	Orange peel and lemon peel (*Citrus spp.* N/A)	1000 W 10 min	N/A	D-limonene yield: 3.7% in orange 2.0% in lemon	[104]
Pectins	Grapefruit peel (*Citrus spp.* N/A)	900 W 6 min	N/A	Pectins yields: 27.81%	[76]
	Orange peel (*Citrus reticulata*)	422 W 169 s pH 1.4 ratio 1:16.9 (g/mL)	Distilled water	Pectins yields: 19.19%	[87]
	Orange peel (*Citrus sinensis* L. Osbeck)	500 W 3 min	Distilled water	Pectins yields: 24.2%	[71]
	Sour Orange Peel (*Citrus aurantium* L.)	700W 3 min pH 1.5 ratio 1:15 (g/mL)	Acidified distilled water	Pectins yields: 29.1% Galacturonic acid: 71%	[105]

Abbreviations: total phenolic compounds, (TPC); galic acid equivalent, (GAE); not available, (N/A); total lavonoids, (TFs); essential oils, (EOs).

**Table 3:** Content of high biological value compounds from citrus wastes extracted with supercritical fluid extraction.

Compound	Sample	Extraction Conditions	Solvent	Results	References
Carotenoids	Mandarin peel (*Citrus unshiu*)	Pressure 150 atm Flow rate 2 mL/min 40 °C 1 h of balance + 1 h of extraction	CO_2_-acetone (7%)	β-carotene: 0.04−0.39 mg/100 g	[114]
Phenolic compounds	Mandarin peel (*Citrus unshiu*)	Pressure 250 atm Flow rate 2 mL/min 60 °C 1 h balanced 30 min extraction time	CO_2_-methanol (7%)	Hesperidin 1.6–1.8%	[94]
	Mandarin, orange, lemon, and grapefruit peel (*Citrus spp.* N/A)	Pressure 160 atm Flow rate 1 mL/min 35 °C	Ethanol 40%	TPC: 0.67 mg GAE/100 g grapefruit 0.66 mg GAE/100 g lemon 0.45 mg GAE/100 g orange 0.38 mg GAE/100 g tangerine	[75]
	Sour orange peel (*Citrus aurantium amara*)	170 bar Flow rate 2.7kg CO_2_/h 50 min balanced time 120 min extraction time	CO_2_-ethanol (3%)	Osthol 47%	[115]
Essential oils	Citron peel (*Citrus medica*)	100 bar Flow rate 5 mmol/min 40 °C 6 h	CO_2_	Oil yield: 28% Citropten: 84.5%	[116]
	Lemon peel (*Citrus lemon*)	15 MPa Flow rate 8 L/min 40 °C 40 min Particle size 0.125–1 mm	CO_2_	D-limonene yield: 4.5%	[117]
	Mandarin peel *(Citrus unshiu*)	Pressure 100 atm 15 °C 15 min of equilibrium	CO_2_	EOs yield: 0.85%	[114]
	Mandarin, orange, lemon and grapefruit peel (*Citrus spp.* N/A)	Flow rate 1 mL/min 35 °C	N/A	Limonene: 3010 mg/100 g mandarin 990 mg/100 g orange 810 mg/100 g grapefruit 70 mg/100 g lemon β-pineno: 39 mg/100 g mandarin 14 mg/100 g orange 10 mg/100 g grapefruit 11 mg/100 g lemon	[75]

Abbreviations: total phenolic compounds, (TPC); gallic acid equivalent, (GAE); not available, (N/A); essential oils, (EOs).

**Table 4:** Content of high biological value compounds from citrus wastes extracted with pressurized water extraction (PWE), pressurized liquid extraction (PLE), and pulse electric fields (PEF).

Method	Sample	Extraction Conditions	Solvent	Results	References
Pressurized water extraction	Mandarin peel *(Citrus inshiu*)	160 °C 10 min 100 atm	Water	Hesperidin 7200 mg/100 g Narirutin 11,700 mg/100 g	[122]
	Mandarin peel *(Citrus reticulata*)	120 °C 5 min Ratio 1:30	Water	Pectin yield: 21.95% Galacturonic acid: 68.88%	[123]
	Orange peel (*Citrus spp.* N/A)	150 °C Flow rate 10 mL/min	Water	TPC: 3170 mg GAE/100 g Hesperidin 2000 mg/100 g Narirutin 233 mg/100 g Pectins yield: 18.53%	[124]
Pressurized liquids extraction	Orange peel (*Citrus spp.* N/A)	65 °C 40 min 10 MPa Flow rate 2.37 g/min Ratio 1:47 kg/kg	Ethanol 75%	TPC: 1590 mg GAE/100 g Hesperidin: 5400 mg/100 g Naringin: 22.6 mg/100 g Narirutin: 42 mg/100 g Tangeretin: 54 mg/100 g Naringenin: 54 mg/100 g Hesperitin: 26 mg/100 g	[125]
Pulse electric field	Orange peel (*Citrus spp.* N/A)	1, 3, 5, and 7 kV/cm 1 Khz frequency 30 min 5 bars	N/A	Phenolic compounds yield: 20%, 129%, 153%, and 159% Antioxidant capacity: 51%, 94%, 148%, and 192% A 5kV/cm 1–3.1 mg/100 g naringin 1.3–4.6 mg/100 hesperidin	[130]
	Orange, grapefruit, and lemon peel (*Citrus spp.* N/A)	First treatment 3kV/cm Second treatment 10 kV/cm	Ethanol 50%	TPC: 2200 mg GAE/100 g orange peel Hesperidin: 507 mg/100 g orange (flavedo) 482 mg/100 g orange (albedo) Naringin: 1036 mg/100 g grapefruit (flavedo) 2686 mg/100 g grapefruit (albedo) Eriocitrin: 144 mg/100 g lemon (flavedo) 197 mg/100 g lemon (albedo)	[131]

Abbreviations: total phenolic compounds, (TPC); gallic acid equivalent, (GAE); not available, (N/A).

**Table 5:** Content of high biological value compounds from citrus wastes extracted with high voltage electric discharges.

Compound	Sample	Extraction Conditions	Solvent	Results	References
Carotenoids	Mandarin peel (*Navel Navelate*)	40kV 10 µs 55 kJ/kg and 364 kJ/kg	N/A	Total carotenoids: 0.369 mg β-carotene/100 mL of extract (55 kJ/kg) 0.286 mg β-carotene/100 mL of extract (364 kJ/kg)	[134]
Phenolics compounds	Grapefruit peel (*Citrus spp.* N/A)	Ratio 1:10 (g/mL) 10–300 pulses	Glicerol (20%)	TPC: 1880 mg GAE/100 g	[135]
	Mandarin peel (*Navel Navelate*)	40kV 10 µs 55–364 kJ/kg	N/A	TPC: 184.2 mg GAE/100 mL of extract (55 kJ/kg) 692.1 mg GAE/100 mL of extract (364 kJ/kg)	[134]

Abbreviations: total phenolic compounds, (TPC); gallic acid equivalent, (GAE); not available, (N/A).

**Table 6:** Content of high biological value compounds from citrus wastes extracted with high hydrostatic pressure (HHP).

Compounds	Sample	Extraction Conditions	Solvent	Results	References
Phenolic compounds	Orange and lemon peel (*Citrus spp.* N/A)	300 MPa 10 min 10 °C 500 MPa 3 min 10 °C	N/A	TPC: 136.85 mg GAE/100 mL orange 344.53 mg GAE/100 mL lemon	[138]
	Lemon, lime, mandarin and orange peel (*Citrus spp.* N/A)	300 MPa	N/A	TPC: 266.23 mg GAE/100 g lemon 397.21 mg GAE/100 g lime 587.28 mg GAE/100 g tangerine 288.16 mg GAE/100 g orange	[19]

Abbreviations: not available, (N/A); total phenolic compounds, (TPC); gallic acid equivalent, (GAE).
